# The Roles of Ubiquitination in Pathogenesis of Influenza Virus Infection

**DOI:** 10.3390/ijms23094593

**Published:** 2022-04-21

**Authors:** Eun-Sook Park, Mehrangiz Dezhbord, Ah Ram Lee, Kyun-Hwan Kim

**Affiliations:** 1 Institute of Biomedical Science and Technology, School of Medicine, Konkuk University, Seoul 05029, Korea; espark97@gmail.com; 2 Department of Precision Medicine, Sungkyunkwan University School of Medicine, Suwon 16419, Korea; m.dezhbord@yonsei.ac.kr (M.D.); ahram2g@naver.com (A.R.L.)

**Keywords:** influenza a virus, post-translational modification, ubiquitination, pathogenesis

## Abstract

The ubiquitin system denotes a potent post-translational modification machinery that is capable of activation or deactivation of target proteins through reversible linkage of a single ubiquitin or ubiquitin chains. Ubiquitination regulates major cellular functions such as protein degradation, trafficking and signaling pathways, innate immune response, antiviral defense, and virus replication. The RNA sensor RIG-I ubiquitination is specifically induced by influenza A virus (IAV) to activate type I IFN production. Influenza virus modulates the activity of major antiviral proteins in the host cell to complete its full life cycle. Its structural and non-structural proteins, matrix proteins and the polymerase complex can regulate host immunity and antiviral response. The polymerase PB1-F2 of mutated 1918 IAV, adapts a novel IFN antagonist function by sending the DDX3 into proteasomal degradation. Ultimately the fate of virus is determined by the outcome of interplay between viral components and host antiviral proteins and ubiquitination has a central role in the encounter of virus and its host cell.

## 1. Introduction

The influenza virus is a globally prevalent human respiratory pathogen, with recurring epidemic and pandemic potentials. Viruses almost entirely rely on their specific host to complete their life cycle and establish infection. These are including several steps such as DNA replication, RNA transcription, expression of vital protein and enzymes, assembly, and budding from infected host cells [1]. Ubiquitination is a vital post-translational modification (PTM) involves in multiple mechanisms inside the cell to maintain its stable condition and homeostasis. Therefore not surprisingly, even tiny errors in the processes controlled by PTM can be devastating and cause serious illnesses [2]. During viral infection, PTM of viral or host proteins, promote virus growth by enhancing viral replication, assembly, and release, and also inhibits host immune activity including interferon-stimulated responses as one of the main antiviral defense strategies. On the contrary, virus infected cells restrain the virus either by removing proviral PTMs and/or degrading crucial viral proteins by adding ubiquitin (Ub) or ubiquitin-like proteins, resulting in the abrogation of crucial viral proteins through proteasome degradation [1].

**Figure 1:**
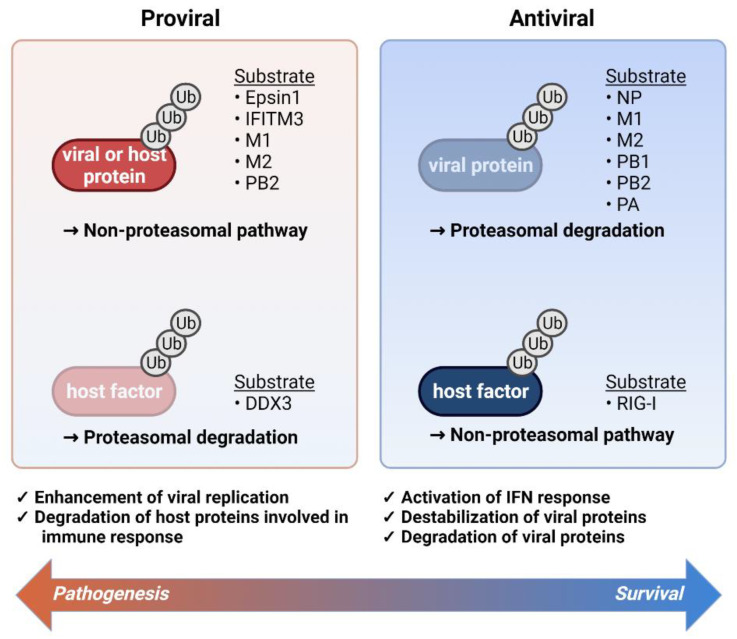
The role of ubiquitination during influenza virus infection. A balance between enhancement and inhibition of viral replication determines the final pathological outcomes of the viral infection. The main viral components are ubiquitinated for proteasomal degradation and RIG-I ubiquitination contributes to the induction of IFN response (**right panel**). On the other hand, host or viral proteins are subjected to the ubiquitination to engage in proviral activity and in parallel, antiviral regulators such as DDX3 ubiquitination suppress immune activation (**right panel**). The viral and host proteins that are targeted by ubiquitination are listed in the **right** (antiviral) and **left** (proviral) side of the figure.

In this review, we summarized the recent knowledge concerning the impact of ubiquitination in influenza virus and its proteins on the virus replication and interplay with host components (Figure 1). We exploit mechanisms used by the influenza virus to ubiquitinate and modulate host protein function in ways that benefit its replication.

## 2. The Ubiquitin System

The cellular ligases (E3), in harmony with ubiquitin-activating enzymes (E1) and ubiquitin-conjugating enzymes (E2), are responsible for the ubiquitination of substrate proteins [3,4]. The human proteome contains two E1 enzymes, approximately fifty E2 enzymes, six hundred E3 ligases [5], and one hundred deubiquitinating enzymes (DUBs) [6,7]. Deubiquitylating (DUBs) enzymes are equally important for the removal of ubiquitin from the target proteins in order to balance the final result of protein tagging [8].

The ubiquitin chains are organized by the conjugation of different ubiquitin lysine residues through variety of linkages. The ubiquitin itself contains seven lysine residues [9,10]. Various types of ubiquitin chains play a role in diverse biochemical signaling, such as membrane trafficking, triggering kinases, and DNA damage response [11,12]. The well-described proteasomal degradation machinery is activated by K48-linked poly-ubiquitination that signals the target protein to the 26S proteasome compartment for proteolysis [13]. The proteasome-independent ubiquitin signaling pathways that recognize K63-linked polyubiquitination and monoubiquitination on the substrate, are equally important for the activation of several biological processes that are vital for the cell.

## 3. Ubiquitination of Viral Proteins

### 3.1. Promotion of Viral Replication

Ubiquitination has been shown to be indispensable for the viral replication, in particular influenza A virus (IAV) [14,15,16,17,18]. Many reports have highlighted the implication of the ubiquitination system in IAV infection (Table 1).

The ubiquitin machinery plays key roles at multiple steps during the complementary effect of the ubiquitination in IAV entry initiates by viral attachment to the host cell that provokes virion entering by means of clathrin-mediated endocytosis [19,35], clathrin-independent endocytosis [36], or macropinocytosis [37,38,39]. The internalization of IAV through clathrin-mediated endocytosis involves Dynamin [35] and requires the ubiquitination of the adaptor protein Epsin 1 [19]. The ubiquitin-interaction motifs (UIM) of Epsin 1 attach to the ubiquitinated virus receptors to facilitate internalization [19].

Interestingly, influenza virus utilizes PTM machinery to ubiquitinate and degrade its entry inhibitors such as IFITM3 by cellular E3 ligase NEDD4 [20]. Nuclear import of influenza ribonucleoprotein (RNP) complex requires efficient un-coating and microtubule network association of the virions. For this reason, non-conjugated ubiquitin chains of influenza virions activate the cellular aggresome where mechanical forces facilitate viral core un-coating and release of RNPs [22,40]. The ubiquitination of viral proteins promotes the viral replication as well. For instance, the influenza PB2 protein is ubiquitinated by the CRL4 E3 ligases through the atypical K29 linkages, which does not result in degradation and rather enhances polymerase action and thus viral replication [25]. Infection-induced cascades elevate the M1 ubiquitination to support release of the newly assembled virions [21]. It has been shown that all RNP components are ubiquitinated, and disturbing ubiquitination hampers RNP function [41]. Influenza nucleoprotein (NP) is actively monoubiquitinated and deubiquitinated at lysine 184 (K184) [27,31] by CNOT4 E3 ligase to enhance the viral RNA-dependent RNA polymerase (RdRp) activity [27]. This modification may control the interaction between NP with RdRp to assist viral genome replication.

In addition, the ubiquitination of IAV matrix protein 2 (M2) enhances viral genome packaging during IAV infection and through facilitating the packaging of viral genome into virus particles and regulating the time points of major host cell processes such as apoptosis and autophagy in a way to benefit the viral propagation [24]. It has been reported that disrupting M2 ubiquitination attenuated the mutant virus due to emerging RNP deficient virus particles [24].

### 3.2. Repression of Viral Replication

Following virus infection, the host cell utilizes ubiquitination machinery to tag and destroy viral substrates and control the virus transmission [18,42,43]. As an example related to IAV, the cellular cyclophilin A ubiquitinates M1 in order to send it for proteolysis [32], and similarly, the viral polymerase subunits PA and PB2 are ubiquitinated by the zinc finger antiviral protein ZAPL which is required for PA and PB2 proteasomal-mediated degradation [33]. Moreover, the tripartite motif-containing (TRIM) family and DUB enzymes are just as important for host defense against IAV infection. Among TRIMs, TRIM14, TRIM22, TRIM32, and TRIM41, retaining E3 ubiquitin ligase activity, directly target IAV proteins, to limit and battle infection [26,28,29,30]. Specifically, the TRIM14 and TRIM41 bind to NP, causing its ubiquitination and degradation, therefore preventing vRNPs’ formation and viral genome replication [26,28]. The TRIM22 has been reported to interface NP to raise its K48-linked polyubiquitination and forward it to proteasomal degradation. Therefore, in human alveolar epithelial A549 cells, the TRIM22 is highly upregulated after viral infection [29].

Nevertheless, removing ubiquitin from NP could affect influenza viral replication. The DUB enzyme USP11 impedes viral RNA (vRNA) replication through removing NP K184 ubiquitin linkage to reduce the NP binding affinity for complementary RNA (cRNA) [31]. The ubiquitin ligase, CNOT4, reverses the action of USP11 and thereby enhances the vRNPs activity and vRNA replication [27]. Thus, the ubiquitination of NP contributes to the outcomes that can be advantageous or dis-advantageous for the virus meaning that the balance of these regulating mechanisms dictates the efficiency of IAV infection.

### 3.3. The Ubiquitination of Influenza Virus Proteins

#### 3.3.1. The Nucleoprotein (NP)

The NP is a structural protein of influenza virus that has a negative-sense RNA genome, and its activity is regulated by ubiquitination. NP has several potential ubiquitination target sites such as K184, K227, and K273, which all reside on the RNA-binding domain of NP [27]. Therefore, it is reasonable to speculate that the monoubiquitination may modulate the RNA binding property of NP. Monoubiquitination of the NP at K184 by the host E3 ligase, enhances viral replication and vRNP activity [31], presumably by the increased RNA binding, cRNA stabilization, and augmented viral RdRp activity (increased transcription and replication by the IAV polymerase) [18,27,44].

Ubiquitination at K184 can also be modulated by the host deubiquitinase USP11 resulting in decreased polymerase activity and reduced viral replication. Although it is obvious that the competition between K184 monoubiquitination activity and USP1-induced deubiquitination activity may determine the final outcome of the RNA binding activity and viral replication by NP, it is not clear how this opposing activity is modulated during viral infection. In addition to the monoubiquitination-induced regulation of its activity, the level of NP can be modulated by polyubiquitination. For example, it has been reported that TRIM14, TRM22, and TRIM41 bind to NP, leading to its polyubiquitination and degradation, which results in the reduced transportation of NP into nucleus, inhibition of vRNPs’ formation, and viral RNA replication [45]. On the contrary, it has also been reported that NP can be polyubiquitinated on other lysine residues by number of E3 ligases, and these modifications are not related to the degradation of NP but other regulations.

Combined with the fact that NP ubiquitination status has both proviral and antiviral roles by monoubiquitination and polyubiquitination, balanced modulation of ubiquitination and deubiquitination of NP may provide a pivotal role for efficient influenza infection.

#### 3.3.2. The Matrix Protein (M1)

The regulatory role of M1 ubiquitination and viral replication by a host factor has been previously described [32]. A RNAi-induced 293T cell line was established with reduced level of endogenous cyclophilin A (CypA), which has been suggested to interact with M1 and reduce viral replication. Viral replication and infectivity were increased with the loss of CypA expression in this condition. Although there were no differences in viral genome replication, transcription and nuclear export of the viral mRNA, levels of M1 protein remains higher in the CypA knockdown cell lines than wild type cell lines, which is mimicked by MG132 treatment in wild type cell lines. Therefore, CypA is responsible for destroying M1 through ubiquitination and this effect could be restored by inhibiting the proteasome.

An alternative role of host factor and the regulation of viral replication has also been suggested. In an effort to systemically screen for cellular factors playing essential roles in IAV replication, Su et al. adopted the genome-wide pooled shRNA screen methods, which identified Itch, an E3 ubiquitin ligase, as a key factor for viral release from endosomes [21]. Itch was shown to interact with influenza M1 protein. M1 exists in multiple ubiquitinated forms and knockdown of Itch reduced the amounts of mono- and oligo-ubiquitinated M1 species. These results suggest that Itch is responsible for the ubiquitination and degradation of M1, which is critical for the translocation of vRNP to the cytoplasm [21]. Thus, the M1 ubiquitination plays an essential role in vRNPs release from endosome [21,27].

The effects of M1 ubiquitination on the final outcome of viral replication might be differentially determined in terms of specific cellular contexts, which remains to be determined in the future.

#### 3.3.3. The Ion Channel Envelop Protein (M2)

Ubiquitination of the M2 protein has been implicated in the regulation of infectious virion production and spreading [24]. It was reported that the ubiquitination of M2 on its K78 residue modulates the packaging of viral genome into virus particles. It has been suggested that ubiquitination at the K78 of M2 induces conformational changes of the particular domain, which facilitates interaction with viral M1. Eventually, this change induces efficient incorporation of vRNA into a new virion [18,24]. In the ubiquitination-deficient mutant M2 K78R, defective virion particles lacking vRNPs or containing smaller amounts of internal viral components were observed, which might be related to lower viral infectivity [24]. K78 ubiquitination of M2 is also involved in the coordination of virus-induced apoptosis and the autophagy process of host cells, which are the essential biological processes regulated by viruses to control infection-mediated cellular death and viral spreading [24,45]. The K78 ubiquitination of M2 may speed up the autophagy induction without affecting the role of M2 to block the completing of autophagy. M2 interacts with HSP40 through CTD1 domain, which further interacts with *p*58 (IPK) to build a firm complex and regulates the activation as well as autophosphorylation of the PKR and [46], which is one of the essential components of host defense response after viral infection. The interaction and activation of PKR eventually induces cell death. A virus carrying the K78R mutation of M2 induced earlier autophagy and apoptosis than WT virus [24], which may result in mistiming of viral pathogenesis and poor viral replication. This might also contribute to the dysregulated host-destruction and occurrence of severe outcomes in patients after infection.

In contrast to the positive role of ubiquitination of M2 on viral replication, it has also been suggested that the membrane-associated RING-CH (MARCH, E3 ligase) induces M2 ubiquitination, which leads to its degradation [47]. A recombinant A/Puerto Rico/8/34 virus encoding a mutant allele, which replaces lysine with arginine to prevent K78 polyubiquitination, showed greater replication activity and more severe pathogenicity both in vitro and in vivo, suggesting the essential role of K78 polyubiquitination and resulting degradation in minimizing adverse outcome after viral infection. Due to the inability of the mutant M2 to be polyubiquitinated and degraded by MARCH8-mediated mechanism, it is suggested as a plausible mechanism for viral evasion of host-induced destruction as well as severe pathogenicity.

#### 3.3.4. The Polymerase Complex (PB1, PB1-F2, PB2, and PA)

The trimeric RdRp complex of IAV is made of three subunits: The PB1(polymerase basic protein 1), the PB2 (polymerase basic protein 2), and the PA (polymerase acidic protein) which binds to each end of the viral genomic RNA. Some of their fragments such as PB1-F2 are subjected to ubiquitination-dependent regulation. For example, an E3 ubiquitin ligase TRIM32 binds to PB1 and induces its K48-linked ubiquitination, which reduces viral polymerase activity [30]. Similarly, it has been suggested that polyubiquitination on PB2 and PA limits the stability of the proteins and reduces the viral polymerase activity [33]. In this case, the antiviral protein ZAPL (PARP-13.1), binds to the mRNAs of some viruses via its N-terminal zinc finger domain and induces mRNA degradation, binding the viral PB2 and PA polymerase proteins through C-terminal PARP and WWE domain, which induces their proteasomal degradation [33]. Interestingly, PB1 polymerase binds near the PARP domain and induces PB2 and PA dissociation from ZAPL, which renders the viral escape against degradation. These observations led the authors to suggest targeting PB1 and ZAPL binding interface for the development of a potential antiviral strategy [33].

In contrast to UPS dependent degradation, ubiquitination of PB2 has been implicated for an optimal IAV infection process [25]. Two Cullin 4 (CRL4)-based multicomponent RING-E3 ligases, one comprising DCAF12L1 and the other one including DCAF11 as substrate recognition factors (SRF), where DDB1 is an intermediate adaptor, play an essential role in the regulation of PB2 ubiquitination and productive viral cycle. The binding of CRL4s to PB2 requires interaction with both the DDB1 adaptor and DCAF substrate receptors, which provides additional regulatory points. It was demonstrated that C-terminal half-region of PB2 contains most of the ubiquitination target lysine residues, which showed similar ubiquitination level as compared with whole length PB2. In contrast, N-terminal half of PB2 did not show significant ubiquitination. Unlike the UPS-activating processes, the CRL4-induced ubiquitination of PB2 primarily consists of atypical K29 linkages, which does not cause its destruction nor the changes in the transcription/replication activity of the polymerase [25]. Compared to typical K48 and K63 ubiquitin signals, K29 type ubiquitination has been implicated in proteasome-independent pathways, such as regulation of Wnt signaling [48], antiviral innate immunity [49], or protein aggregation [50]. Most importantly, recombinant viruses harboring mutations in the lysine target residues resulted in less virus growth, implying that CRL4-dependent ubiquitination of PB2 promotes IAV infection. In addition, virus production was decreased upon silencing of the CRL4 and increased by the ectopic expression of DDB1 and DCAF11 [51]. Although the exact mechanism of the proviral effects of CUL4-mediated PB2 ubiquitination is not reported, the authors demonstrated the interaction of the CRL4 E3 factors with the trimeric viral polymerase containing PB1-PA pair through PB2 using GPCA-based quaternary protein complex experiments [25]. These findings indicate that the ubiquitination of PB2, PB1, PA, and NP plays a proviral role independent of their proteosomal degradation [41]. The ubiquitination increases polymerase activity and interestingly enough MG132 treatment to inhibit proteosomal activity which hampers the viral mRNA transcription, the replication intermediate cRNA, and the final vRNA suggesting the essential role of proteosomal activity in the viral infection although there is no observable viral polymerase complex proteosomal degradation upon ubiquitination [41].

In addition to conventional IAV viral proteins, several accessory proteins such as PA-X and PB1-F2 were discovered, which can regulate the host innate immunity and the adaptation of influenza to mammalian hosts. Of particular interest is PB1-F2, which is a protein consisting of ninety amino acids and encoded from the PB1 ORF in some influenza viruses. PB1-F2 has been engaged in the modulation of apoptosis and cell death [52], host innate immune response [53], and viral polymerase activity [54], thereby contributing to the pathogenesis and comorbidity of influenza such as the secondary bacterial infection [17,55]. During the virus replication, the mitochondrial inner membrane space is targeted by PB1-F2 which dysregulates the RLR-dependent antiviral signaling by attaching to mitochondrial antiviral signaling protein (MAVS) and interfering with IFN expression [56,57,58]. On the other hand, formation and accumulation of highly cytotoxic amyloid-like fibers of PB1-F2 protein was reported after IAV infection which might underlie the immunopathological disorders observed during IAV infections [59].

Ubiquitination-induced PTM of PB1-F2 were identified as a crucial event for the control of viral RdRp activity [44,60]. Loss of PB1-F2 ubiquitination by changes of C-terminal lysine to arginine mutagenesis results in the deubiquitination of PR8-derived PB1-F2 and increases the nuclear localization of PB1-F2, polymerase activity, IFN-beta antagonism and induced higher antibody responses, while the WT showed weak antibody reaction [60]. Similar mutational changes in the C-terminal lysine residues were reported for both avian A/Honk Kong/156/1997 (H5N1)- and mammalian A/Puerto Rico/8/1934 (H1N1)-derived PB1-F2, which are the two evolutionary distinct forms, suggesting a conserved role of lysine residues in the modulation of PB1-F2 stability in virus-host interaction.

Recently, we identified that the 1918 PB1-F2 (PB1-F2 sequence of 1918 influenza pandemic), one of the immunopathology enhancers of the 1918 influenza pandemic [61], is subjected to extensive ubiquitin-dependent proteolysis. In addition to the interference of mitochondria-dependent pathway of type I IFN signaling, 1918 PB1-F2-induced targeting of the DEAD-box helicase DDX3, an important downstream mediator in IFN signaling, to proteasomal degradation pathway could be a basis for high virulence of the 1918 pandemic influenza [62]. The protein-protein interaction analysis revealed that the 1918 PB1-F2, but not PR8 PB1-F2, binds to and down-regulates DDX3. The recombinant DDX3 completely rescued the mice from fatal IAV (1918) infection with improvement of IFNβ induction and reduction of the lung viral titer [62]. These results suggest that ubiquitin-proteasome pathways not only directly regulate the protein activity/level of viral components but can also co-modulate essential host factors by sequestering them into the UPS pathway by interacting with PB1-F2. Identification of other PB1-F2 interacting host factors, as well as the specific role of PB1-F2 molecules in this context awaits further investigation in order to target this mechanism for the development of new antiviral therapeutics.

#### 3.3.5. The Nonstructural Protein 1 (NS1)

NS1 is a nonstructural viral protein which is generally composed of 230 amino acids, although its length may vary due to the mutations affecting the availability of stop codons. It is generally involved in the regulation of host translational machinery as well as polyadenylation steps for mRNA mediating inhibition of host innate immune responses, such as IFN production [63,64]. One of the key regulators of the IFN production is TRIM25-RIG-I signaling pathway, a vRNA sensor. It was demonstrated that the polyubiquitination on RIG-I CARD domain with K63 polyubiquitin chains induced by TRIM25 led to the IFN production. NS1 binds to and suppresses both human TRIM25 and Riplet E3 ligase mediated RIG-I ubiquitination, which results in perturbation of the IFN production. Instead of affecting TRIM25 expression level, NS1 interacts with the CCD moiety of the TRIM25 and revokes TRIM25 multimerization and enzymatic activity, which is essential for ubiquitination on RIG-I [65]. Besides, the inhibition of IFN production seems to be mediated by the prevention or disruption of the formation of RIG-I/MAVS complexes [17,65,66,67].

Furthermore, other pathways leading to type I IFN production, such as TLR3 and TLR7, are modulated by the NS1 through targeting the TRAF3 E3 ubiquitin ligase. NS1 binds to and suppresses TRAF3 K63-linked ubiquitination, resulting in the inhibition of IFN genes expression [68]. NS1 carries a conserved FTEE motif (a.a. 150–153) and glutamate residues at positions 152 and 153 that have been suggested to be critical for binding to and blocking ubiquitination of the TRAF3 in order to suppress type I IFN production. Mutation of these essential glutamate residues into alanine renders more type I IFN production in vitro and in vivo, and the infected mice with the recombinant virus exhibit the attenuated phenotype, suggesting the essential role of NS1-regulated TRAF3 modulation antagonizing host interferon response.

Recently, it has been suggested that the NS1 can modulate JAK-STAT signaling by epigenetic modifications, in this case, the DNA methylation of the promoter regions of its key regulators. It was reported that the NS1 exclusively interacts with the DNA methyltransferase 3B (DNMT3B), which dissociates the DNMT3B from the JAK-STAT regulatory genes promoters. Additionally, the binding of NS1 with DNMT3B transports it to the cytosol and promotes the K48-linked ubiquitination and degradation of DNMT3B [69], which may further reduce DNA methylation on the genes of major JAK-STAT components. These results suggest that the NS1 can modulate epigenetic mechanism of the host immune response, at least in part by altering the cellular location of epigenetic regulators as well as ubiquitination-mediated proteasome activity, which can be studied as a potential target for therapeutic intervention.

#### 3.3.6. The Hemagglutinin (HA)

Not only involved in the control of infectivity of influenza virus, the influenza hemagglutinin (HA) can induce ubiquitination on the key molecules of IFN producing pathways. IAV induces the phosphorylation and ubiquitination of IFNAR subunit 1 (IFNAR1) which results in degradation of IFNAR1 and attenuation of type I IFN responses [70]. The IAV hemagglutinin, especially HA1, was demonstrated to switch on the ubiquitination of IFNAR1, eliminating the expression of IFNAR1 without affecting IFNAR1 mRNA level nor modulating the endoplasmic reticulum (ER) stress response. HA-induced ubiquitination and decreased expression of IFNAR1 is another evasion mechanism adapted by the virus to avoid the host immune system, on top of the already reach repertoire adopted by the virus.

#### 3.3.7. The Neuraminidase (NA)

The influenza neuraminidase (NA) was initially identified as a surface protein essential for virus release [71]. More studies revealed its further role in the early steps of influenza life cycle, mainly at the virus entry. The glycopeptide MS analysis have shown that NA, as a glycoprotein, undergoes glycosylation PTM not ubiquitination by host cell factors [72].

## 4. Ubiquitination of Host Proteins

### 4.1. Promotion of Host Innate Immune Response

Innate immune responses are the primary defense mechanism against pathogens [73]. It begins with the recognition of pathogen-associated molecular patterns (PAMPs via pattern-recognition receptors (PRRs) [74]. Regarding the host antiviral immunity, after virus-derived PAMPs identification, PRRs activate different intracellular signaling cascades, which leads to the production of type I IFNs as well as pro-inflammatory cytokines. IFNs are pivotal for the expression of various interferon-stimulated genes (ISGs). The proteins and enzymes produced from these ISGs eventually inhibit and control viral infection and create the adaptive immunity [75]. PTMs interplay with antiviral processes by regulating the main components of these signaling pathways [76].

#### Retinoic-Acid-Inducible Gene-I (RIG-I)

**Figure 2:**
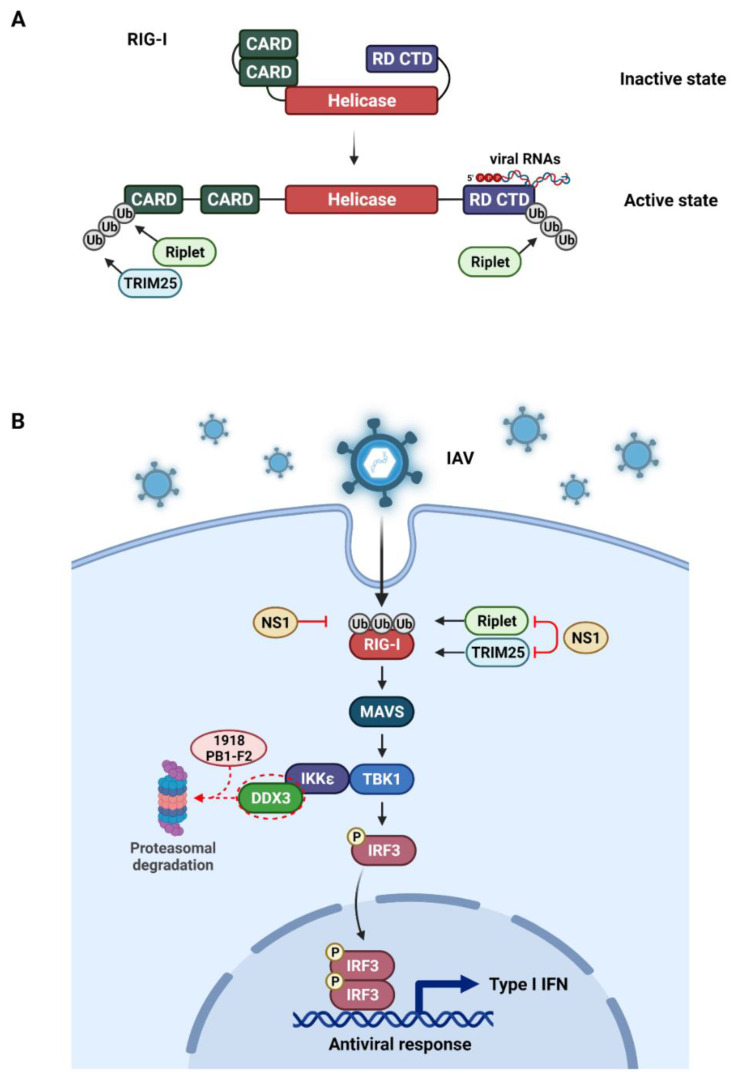
The influenza virus evasion mechanism through ubiquitin modulation of host cell proteins. The IAV recognition signaling pathway, triggered by the innate immune sensor RIG-I represented. (**A**). The RIG-I binds to the viral RNA (e.g., the 5′ppp RNA) and undergoes a conformational change that opens CARD domains. The CARD domain is then activated by ubiquitination, catalyzed by either TRIM25 or Riplet E3 ligases. (**B**). After viral RNA detection, RIG-I recruits MAVS to activate the TBK1-IKKϵ complex, which is needed for the activation of IRF3. The IRF3 then translocates to the nucleus and induces type I IFNs. The IAV NS1 suppresses the TRIM25 or Riplet-mediated ubiquitination of RIG-I caspase. The NS1 RBD from 1918 H1N1 strain inhibits RIG-I ubiquitination. Highly pathogenic PB1-F2 protein from 1918 H1N1 strain also antagonizes IFN production by proteasomal-mediated degradation of DDX3. This illustration was created with BioRender.com.

RIG-I is a significant viral RNA sensor that specifically identifies double-strand viral RNA (dsRNA) and has been shown to be especially activated during IAV infection [77,78]. RIG-I is a RNA helicase with two N terminal caspase activation and recruitment domains (CARDs), a main helicase domain, and a C-terminal domain (CTD) [79] (Figure 2A). These domains are required binding to the RNA and MAVS as downstream adaptors [76].

The ubiquitination machinery conducts the PTM on RIG-I once it is bound to the substrate. Specifically, after disclosure of the CARDs, RIG-I is K63 poly-ubiquitinated at Lys172 by TRIM25 [80,81]. Furthermore, RIG-I is ubiquitinated by other E3 ligases such as Riplet (RNF135 or REUL) and several sites within the CARD or CTD domain such as Lys788 are essential for its ubiquitination [82,83,84]. The interaction between ubiquitinated RIG-I and MAVS ultimately induce the type I IFN production [77].

### 4.2. Evasion of Host Innate Immune Response by Viral Proteins

The RIG-I activation is closely regulated by diverse PTMs in order to hold back the aberrant innate immunity. However, viruses have developed multiple adaptive strategies [85,86,87], such as hiding viral components from RIG-I sensor and destroying receptor or signaling proteins (Figure 2B). This interesting virus–host interplay has extended our knowledge of viral pathogenicity and function of the RIG-I pathway [88].

For instance, the IAV NS1 binds to and impedes both human TRIM25 and Riplet-mediated RIG-I ubiquitination, which results in perturbation of IFN production [89]. Instead of affecting TRIM25 expression level, NS1 interacts with the central coiled-coil domain (CCD) of TRIM25 and abolishes TRIM25 conformational change and enzymatic activity, which is essential for ubiquitination on RIG-I CARD [65,90] (Figure 2B). The inhibition of IFN production seems to be mediated by the prevention or disruption of the formation of RIG-1/MAVS complexes [17,65,66,67]. The NS1 of different strains of IAV prevents RIG-I ubiquitination and activation by suppressing TRIM25 internal domain interaction, which is a prerequisite to mediatings RIG-I ubiquitination [65,91,92]. Besides, a recent study demonstrated that NS1 RBD from 1918 H1N1 strain directly attaches to the RIG-I CARD domain and blocks its ubiquitination [93]. NS1 mutation reverses this inhibitory function, and results in a higher immune response [93,94].

Similarly, the non-structural IAV PB1-F2 is capable of dysregulating the innate immune responses, by inhibiting type I IFN induction and targeting MAVS [57,95]. Yinping Du et al. reported that IAV by ubiquitin mediated degradation of JAK1 could inhibit IFN type I and II as a mechanism of evasion [96]. Recently, we identified that the highly pathogenic PB1-F2 protein from 1918 H1N1 strain directs DDX3 to proteasomal degradation and therefore antagonizes the IFN pathway [62] (Figure 2B). The interaction between influenza NS1 and NP and DDX3 was shown to exert antiviral activity by regulation of stress granule formation [97]. The reduction of DDX3 via proteolysis inhibited IRF3 phosphorylation and effectively protected the virus from the IFNβ response explaining the enhanced virulence of 1918 pandemic. Besides the NS1 protein, 1918 PB1-F2 constitutes a powerful IFN antagonist, resulting in the severity of the 1918 influenza strain.

## 5. Roles of Ubiquitination in Pathogenesis of Influenza Virus Infection

The pathogenesis of influenza virus infection is mostly due to the increased viral replication or inhibition of cellular antiviral pathways. Although there have been numerous studies on the host or viral protein-induced antiviral strategies, perturbation of the cellular ubiquitin system is the most common and well-known mechanism.

The most devastating IAV infection in human history was the 1918 pandemic, which caused staggering deaths worldwide [98,99]. Although the molecular mechanism of extremely high pathogenicity of the 1918 pandemic has been controversial, recent studies have reported that a specific protein in the virus is attributable to its high virulence [100,101].

Among various viral proteins, the mutated PB1-F2 (1918 PB1-F2) has been suggested to be involved in pathogenesis of the 1918 pandemic [62]. The wild type PB1-F2 (or PR8-derived PB1-F2) also strongly dysregulates innate immune responses by targeting the mitochondria [57,95]. The wild type PB1-F2 contributes to pathogenesis by activation of the NLRP3 inflammasome [102]. However, the extremely high pathogenic nature of 1918 IAV infection is due to the mutation in 1918 PB1-F2 protein [62].

During the 1918 pandemic, aside from the primary viral infection, the secondary bacterial infection severely contributed to death rates. However, the main reason for the high susceptibility of the 1918 IAV infected patients to the secondary bacterial infection remained unclear until it was reported that 1918 PB1-F2 is implicated in the unique immunopathology and enhances inflammatory responses in the lung through enhanced expression of 1918 PB1-F2 as compared to the WT PB1-F2 [100,103,104,105,106].

It has been previously demonstrated that substitutions in the amino acid sequence of PB1-F2 protein contribute to its stability and functions [107,108]; however, the underlying mechanism was not elucidated. Recently, we identified that the 1918 PB1-F2 is subject to extensive ubiquitin-dependent proteolysis [62]. We showed that the extremely low stability of 1918 PB1-F2 is related to the suppression of type I IFN signaling. The interactome analysis revealed that 1918 PB1-F2 binds to the host DDX3, a central mediator in antiviral interferon pathway. Surprisingly, the wild type PB1-F2 did not bind to DDX3. Since 1918 PB1-F2 has been rapidly degraded by the proteasome. The 1918 PB1-F2 bound DDX3 was also rapidly co-degraded, resulting in very low protein stability. Therefore, 1918 PB1-F2 protein exerts a novel IFN antagonist function by sending the DDX3 into proteasomal degradation, thereby depleting the antiviral interferon signaling (Figure 2B). The virus harboring the mutated 1918 PB1-F2 showed the enhanced viral replication and postponed clearance of the virus in mice lungs, which may have contributed to the enhanced virulence of the 1918 pandemic.

Furthermore, the sequencing analysis showed that there were 12 amino acid substitutions in 1918 PB1-F2 protein compared to the wild type PR8 PB1-F2. To demonstrate how only the mutated 1918 PB1-F2 interacts with DDX3, we conducted the mutational analysis and found that mutations I68T and L69P of 1918 PB1-F2 are determinant for DDX3 binding and crucial for the increased pathogenicity in mice.

These findings prompted us to identify those mutations in IAVs isolated over the last decade. First, more than 80% of the human-infecting H1N1 IAV isolates showed the truncation of PB1-F2 protein, whereas the majority of the avian H1N1 isolates retained its full ORF [109]. No L69P mutation was found in all human IAVs samples over the past decade, even viruses carrying the entire PB1-F2. Notably, the L69P mutation in PB1-F2 has been unique to the 1918 virus until the most recent circulating IAVs. These studies emphasize the critical roles of ubiquitination of viral proteins in the pathogenesis of influenza virus infection and unravel the molecular basis of the highly pathogenic 1918 IAV.

## 6. Conclusions and Perspectives

Ubiquitin modifications of viral and host proteins are vital to the regulation of host responses and survival of viruses. The ubiquitination supports the host to combat viral infection by inducing the immune system and destroying viral components and playing a proviral function where they contribute to assembly, enhancing the viral protein activity and blocking the IFN response. Ultimately, the outcome of the disease is decided by superiority of host immunity over viral growth and vice versa.

As a perspective regarding the ubiquitination of influenza virus proteins, since the I68T and L69P mutations in 1918 PB1-F2 induce active ubiquitin-dependent proteasomal degradation which are coupled to severe pathogenicity, close monitoring of PB1-F2 mutations could contribute to predicting the next highly pathogenic virus that will become a global pandemic in the future. Furthermore, by modulating the ubiquitin pathway, it will help develop novel alternatives and antivirals for the highly pathogenic influenza pandemic.

## Figures and Tables

**Table 1:** Roles of ubiquitination in the influenza A virus infection.

	Major Life Cycle Event	Host Cell Protein	Ubiquitinated Proteins	Outcoming Response	Reference
	Entry/Internalization	Epsin1	Epsin 1	Important for clathrin-mediated endocytosis of virus	[19]
	Entry/Fusion	NEDD4	IFITM3	Promotes viral fusion	[20]
	Entry/Uncoating	ITCH	M1	Ensure efficient uncoating	[21]
Proviral		HDAC6	Virion	Ensure efficient uncoating	[22,23]
	Assembly/Packaging	Unknown	M2 at K78	Facilitates the packaging of the viral genome into virus particles	[24]
	Viral RNA replication	CRL4	PB2	Enhances polymerase action and thus viral replication	[25]
	Entry/Nuclear import of vRNPs and vRNPs components	TRIM14	NP	Decreases NP nuclear import	[26]
	Viral RNA replication	CNOT4	NP	Enhances replication by serving as competing enzymes with USP11	[27]
Antiviral		TRIM41	NP	Limits virus infection	[28]
		TRIM22	NP	Limits virus infection	[29]
		TRIM32	PB1	Inhibits the activity of RNA polymerase	[30]
		USP11	Deubiquitinating on K184 of NP	Inhibits viral RNA replication	[31]
		Cyclophilin A	M1	Inhibits viral RNA replication	[32]
		ZAPL	PB2, PA	Inhibits viral RNA replication	[33]
	Entry/Assembly	MARCH8	M2 at K78	Limits virus infection	[34]

## Data Availability

Not applicable.
